# Simplifying gene trees for easier comprehension

**DOI:** 10.1186/1471-2105-7-231

**Published:** 2006-04-27

**Authors:** Paul-Ludwig Lott, Marvin Mundry, Christoph Sassenberg, Stefan Lorkowski, Georg Fuellen

**Affiliations:** 1 Division of Bioinformatics, Biology Department, University Münster, Schlossplatz 4, 48149 Münster, Germany; 2 Institut für Informatik, Fachbereich Mathematik und Informatik, Einsteinstr. 62, 48149 Münster, Germany; 3 Department of Medicine, AG Bioinformatics, University Münster, Domagkstrasse 3, 48149 Münster, Germany; 4 Leibniz-Institute of Arteriosclerosis Research, University Münster, Domagkstrasse 3, 48149 Münster, Germany; 5 Institute of Biochemistry, University Münster, Wilhelm-Klemm-Str. 2, 48149 Münster, Germany; 6 Institute of Mathematics and Computer Science, University Greifswald, Jahnstrasse 15a, 17489 Greifswald, Germany

## Abstract

**Background:**

In the genomic age, gene trees may contain large amounts of data making them hard to read and understand. Therefore, an automated simplification is important.

**Results:**

We present a simplification tool for gene trees called TreeSimplifier. Based on species tree information and HUGO gene names, it summarizes "monophyla". These monophyla correspond to subtrees of the gene tree where the evolution of a gene follows species phylogeny, and they are simplified to single leaves in the gene tree. Such a simplification may fail, for example, due to genes in the gene tree that are misplaced. In this way, misplaced genes can be identified. Optionally, our tool glosses over a limited degree of "paraphyly" in a further simplification step. In both simplification steps, species can be summarized into groups and treated as equivalent. In the present study we used our tool to derive a simplified tree of 397 leaves from a tree of 1138 leaves. Comparing the simplified tree to a "cartoon tree" created manually, we note that both agree to a high degree.

**Conclusion:**

Our automatic simplification tool for gene trees is fast, accurate, and effective. It yields results of similar quality as manual simplification. It should be valuable in phylogenetic studies of large protein families. The software is available at .

## Background

The large number of ongoing genome and EST (expressed sequence tag) sequencing projects provides a massively increasing amount of protein data. The physiological role and function of almost all proteins identified by these projects is not known. Due to the large number of newly identified proteins, it is not possible to annotate their function using conventional experimental approaches or manual data analysis, as demonstrated by any paper reporting a newly sequenced organism. Since the 1970s numerous tools have been developed in order to perform automated analyses of sequence data and to annotate functions of proteins based on these analyses. The interpretation of results derived from these automated analyses is also a daunting task due to the complexity of the information revealed.

Phylogenetic analyses of large protein datasets are of prime importance because these analyses often yield better functional predictions than homology searches [1-3]. Moreover, they yield insight into the evolution of single proteins or protein families. Therefore, large amounts of phylogenetic data have been generated and analyzed in recent years. The aim of these studies was, for example, to investigate microbial evolution [4] or to predict the physiological function of experimentally uncharacterized proteins [1].

Sicheritz-Ponten and Andersson [4] automated phylogenetic analysis of microbial genomes in a tool called *pyphy*. They introduced crude tree structure schemata called „phylogenetic connections". Using these, for each gene in a bacterial genome the users of *pyphy *can then determine e.g. whether it features nearest neighbors only from the archaeal kingdom. Given the set of gene trees calculated for the genes of an organism (called "phylome" in their paper), it would be worthwhile to postprocess these to check whether genes in certain subtrees were subject to gene duplication, or not. For example, the subtree of the archaeal neighbors of a bacterial gene may just feature diversification according to species phylogeny, without duplications, and be simplified accordingly.

Frickey and Lupas [5] describe an automated "phylome generation and analysis" tool that is inspired by pyphy and includes improvements on the generation of the multiple alignments. Moreover, they maintain a database of the gene trees constructed and enable extraction of all phylogenies that match specific constraints on tree structure. Presenting the entire database as well as specific extracts to the user would benefit from a tree postprocessing step that makes trees more digestible by the human expert.

Gouret et al. [6] report an "intelligent automation of genomic annotation" which calculates gene trees, guided by an expert system. For each protein family, three different phylogeny reconstruction methods are used, and a consensus is calculated. Again, postprocessing steps that simplify all these trees would be valuable.

Fuellen et al. [1] generated a large tree of 1,138 ABC (ATP-binding cassette) protein sequences, which they investigated for evolutionary patterns of function and domain arrangement. In a major manual effort, they produced a simplified "cartoon tree" which was then annotated by function and domain arrangement.

In summary, there is a strong demand to ease the analysis of phylogenetic data. Specifically, simplifying gene trees to their essential core information content is of maximum importance. The *TreeSimplifier *tool described in this paper has been designed in response to this need of generating "cartoon trees" from large gene trees. It relies primarily on the simplification of those parts of the gene tree that follow species phylogeny. The main features of the software are described in Table 1. Overall, gene trees are compressed in a way that is meaningful, fast and effective.

### Implementation

The main components of our *TreeSimplifier *tool are the following:

• A parser for a gene tree in standard Newick [7] format, with labels that include HUGO (Human Genome Organization) gene classification codes [8] and species designations.

• A set of formal rules to reduce the complexity of the gene tree.

• A graphical user interface that allows the user to customize and execute the rules, to visualize the result, and write it to a file.

TreeSimplifier is designed as an open framework and it is distributed as Java Open Source. New rules can easily be integrated. We now describe each component in turn.

### Gene tree parsing

The input file format for the *TreeSimplifier *application is a gene tree represented by a bracketed expression in Newick format, e.g. ((gene1, gene2), gene3) in the simplest case. More generally, the Newick format describes a species or gene tree by representing the tree structure as pairs of corresponding parentheses around leaf labels that represent the species or genes. Our simplification rules rely on HUGO gene name nomenclature and auxiliary species tree information. Therefore, the leaf labels representing the genes include the following information (see Table 2 for examples).

• The gene name, enclosed by pipe ("|") symbols (mandatory).

• The species designation, enclosed by square brackets (mandatory).

• Optionally, a user-defined discriminator, enclosed by curly braces.

Useful tree simplification is possible if at least some of the gene names follow the HUGO gene classification system. The gene tree may also include bootstrap values and branch lengths, following Newick format. The discriminator can be used to control the simplification process on a leaf-by-leaf basis. In particular, the user can instruct the software to block the execution of simplification rules in those parts of the gene tree where the discriminator of the leaves does not match. An example for the use of the discriminator is given in the Results section on ABC proteins [see appendix 1].

Our leaf label specification follows standard definition line (defline) format, as can be seen from the examples in Table 2, so that trees automatically generated from sequences found in databases like the NR (non-redundant) database [9] can be processed easily. Our parser uses regular expressions to recover the necessary leaf information from the leaf labels. There are three regular expressions. Expression 1 matches the gene name, expression 2 matches the species designation, and expression 3 matches the discriminator (if any). In each case, only the first match is considered. If a leaf label includes, say, two gene names enclosed by pipe symbols, the second gene name is ignored. Matched parts of the leaf label are highlighted in the examples of Table 2.

Our tool is designed to process a gene tree including only genes from a single protein family. For this protein family, the HUGO stem symbol must be known [8]. This symbol is an alphanumerical string that is associated uniquely with the protein family. The stem symbol is considered to be case-insensitive, permitting e.g. use of lower case for mouse genes. It is needed as part of the configuration input. In abstract terms, for a stem symbol *xxx*, a typical HUGO gene name is *xxxA1a*. For concrete examples, see the ABC protein example below, part **(1)**. For a given HUGO gene name such as *xxxA1a*, the stem symbol enables identification of the start position of the hierarchical *gene classification *(on the subfamily, subsubfamily, etc., that the gene belongs to). This HUGO gene classification is denoted by a string of letters and numbers that follow the stem symbol. To parse the gene classification, the stem symbol is deleted from the gene name. Then, the remainder is read step-by-step, going deeper in hierarchy whenever a switch from an alphabetical character to a numerical character is encountered. For some genes, in particular non-human ones, or newly discovered ones, the gene classification may be missing (there may not even be a stem symbol) or incomplete. (See the ABC protein example below, part **(2)**.)

### Genes classified consistently

**Figure 1:**
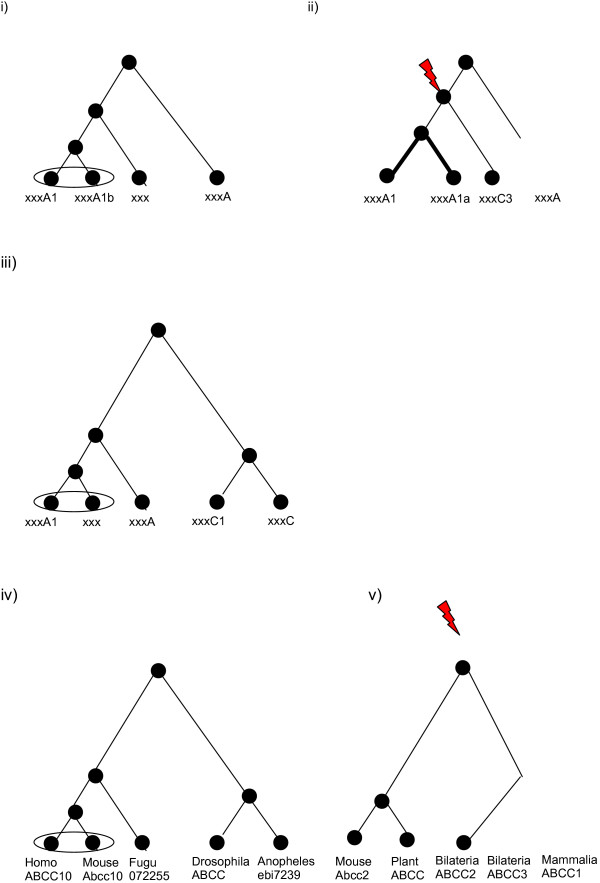
**Consistent and inconsistent classification of genes**. i) A subtree of a gene tree classified consistently. The stem symbol here is "xxx". ii) A subtree (shown in bold) that is classified consistently, and that is part of a larger tree not classified consistently. The conflict occurs at the node marked by the zigzag symbol. iii) An example of a subtree that features two subfamilies. iv) An example of a subtree of ABC genes classified consistently. v) An example of a subtree of an ABC gene tree not classified consistently. Again, the zigzag symbol marks the conflict.

Our approach is motivated by gene trees that feature subtrees with a mix of (A) genes with a HUGO-based gene classification and (B) genes where the classification is missing or incomplete. For examples, see Fig. 1. Genes in a subtree of the gene tree have a *consistent classification *if all gene classifications are prefixes of the longest gene classification found in the subtree, as in Fig. 1, panel i. Here, "xxx" is the stem symbol, and xxxA1, xxxA1b, xxx and xxxA are all prefixes of xxxA1b. If a subtree features only consistently classified genes, it is called a *consistently classified *subtree. In a consistently classified subtree, a gene name that is missing classification information completely or partially (e.g. only the subfamily but not the subsubfamily is given) is assumed to feature the longest classification. A consistently classified subtree may be of maximum size. Then, it cannot be extended without including gene names with an inconsistent gene classification that is not a prefix of the largest gene classification in the subtree. In Fig. 1, panel ii, the subtree marked in bold is consistently classified and of maximum size. For real-world examples, see the ABC protein example below (part **(3)**).

If a subtree is not classified consistently, there are two possibilities. Firstly, the subtree may indeed feature two distinct subfamilies or subsubfamilies. An example for a subtree with two distinct subfamilies A and C can be found in Fig. 1, panel iii. Secondly, we may have identified misplaced or misnamed genes in the gene tree. Distinguishing these cases is not automated, and correct interpretation of the situation is left to the user. In case of Fig. 1, panel ii, it is possible that xxxC3 is a misplaced or misnamed gene. Evidence for this interpretation is that the genes in the subsubtree marked in bold as well as the gene *xxxA *at the root of the entire subtree are members of the A subfamily. Alternatively, however, gene *xxxA *may be misplaced or misnamed. For a real-world example, see the ABC protein example below, part **(4)**. By default, genes (or gene fragments, see below) with a different discriminator cannot be classified consistently. Only subtrees that feature genes with a consistent classification are *candidates *for simplification into single leaves by the compression rules to be described.

The user can specify that the classification is *ignored *from a specific level onwards. For example, the tool can be instructed to classify consistently all genes that share the same subsubfamily, ignoring the next level. (See the ABC protein example below, part **(5)**.) On the other hand, it is up to the user to fine-tune the a-priori classification information given by the HUGO gene names, manually adding further levels that are known. The more detailed the classification, the more exact the identification of misplaced or misnamed sequences, but the fewer simplification can take place. Usually, the best compromise is to stick to the classification implicit in the HUGO gene names, which is well established so that it is unlikely to label genes as misplaced that are in fact placed correctly, and which allows for a high degree of simplification. In specific cases, e.g. in detailed investigations of a single subfamily, or in global analyses of a large gene family, it may be necessary to adjust the degree of resolution.

### Species tree

For gene tree simplification, we need a species tree of all the species that contribute genes to the gene tree. The gene tree can be provided by the user, or the tool uses the NCBI taxonomy [9].

### Gene tree simplification

We will describe three rules for simplifying gene trees. These are a formalization of the rules described in the appendix [see appendix 2]. Some concepts adapted from phylogenetic systematics are defined along the way. Gene trees are assumed to be bifurcating and rooted. The *cenancestor *of a group of species is the most recent common ancestor, also known as "least common ancestor". The cenancestor of a bifurcation in a gene tree is the cenancestor of the species found in the labels of its subtrees. To ease the description that follows, the cenancestor of a single species is the species itself. The consistent classification (see above) of a bifurcation is considered to be defined if the genes in its subtrees are classified consistently. If it is defined, it is the longest classification found in the subtrees.

### Rule 1. Monophyletic compression (Fig. 2)

**Figure 2:**
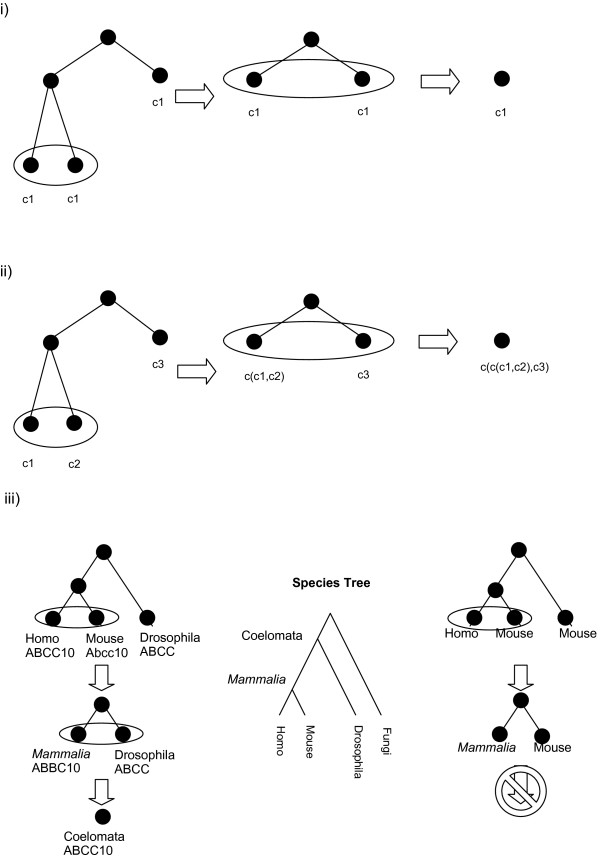
**Step-by-step monophyletic compression**. i) Compression of single-species leaves. Here, c1 must be labeled by a single species, not a group of species. ii) Compression involving group leaves. In the species tree, c1 and c2 must refer to different subtrees of the species tree. In the last step, c(c1;c2) (the cenancestor of c1 and c2) and c3 must also refer to different subtrees of the species tree. Compression of single-species leaves may be followed by compression involving group leaves, but not vice versa. iii) Sample cases from the ABC protein example. The species tree is in the middle. Compression is possible on the left panel but it stops on the right panel. *Mammalia *and mouse are referring to the same subtree of the species tree, since *mammalia *include mouse. Note that *mammalia *is a group leaf.

This rule uses species information to summarize those parts of the gene tree where a subtree follows species phylogeny. A subtree follows species phylogeny if its branching pattern does not contradict the species tree. That is, the subtree features a single gene that evolved according to species phylogeny. The gene does not need to be present for each species in the species tree. Moreover, gene duplications *after *the last speciation event in some part of the subtree are allowed, as in Fig. 2, panel i. Here, a gene from a single species c1 duplicated. Simplification into a single node is possible if the classification of the gene names in the subtree is consistent. Obviously, such a simplification cannot contradict the species tree. Fig. 2, panel ii covers the standard case where a single gene found in species c1, c2 and c3 evolved according to the species phylogeny ((c1,c2),c3). This case may be considered to be different from the case where a gene has duplicated, but the first copy still exists in species c1 and c2, and is lost in species c3, whereas the second copy is lost in c1 and c2, and still exists in c3. However, as long as the classification is consistent, both cases cannot be distinguished. Therefore, in both cases, we will simplify the subtree to a single node, assuming that the gene evolved according to species phylogeny.

Let a species tree of all species be given. We will simplify the gene tree step-by-step, beginning at the *single-species leaves*. We will replace bifurcations of single-species leaves by single leaves, if the classification is consistent. A new leaf is labeled by the cenancestor and the consistent classification of the bifurcation it replaces. If a new leaf involves more than a single species, we call it a *group-of-species leaf ( *or a *group leaf *for short). We will also replace bifurcations involving group leaves if they do not contradict the monophyletic groupings given by the species tree, nor the classification.

In general terms, traversing the gene tree towards the root, only monophyletic bifurcations are replaced. A bifurcation is *monophyletic *if its two outgoing edges are either *i) *leading to two *single-species lea*ves classified consistently, or *ii) *leading to two leaves classified consistently and labeled by two cenancestors that are in distinct subtrees of the species tree. If the two cenancestors are in distinct subtrees of the species tree, species phylogeny is followed. If the two cenancestors are in the same subtree of the species tree, a duplication must be assumed to match the gene phylogeny with the species phylogeny. An example involving ABC proteins is mentioned below, ABC example part **(6)**.

**Figure 3:**
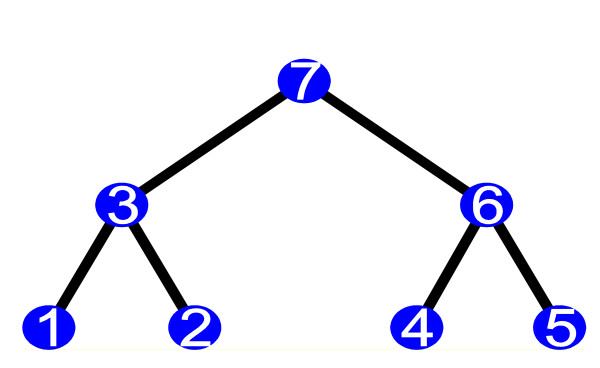
Post-order traversal of a sample tree, following the nodes in numerical order.

As described, the label of a new leaf consists of the cenancestor of the two leaves deleted, and their consistent classification, that is the more detailed one of the two classifications of the leaves deleted. (If both classifications are missing, the first gene name is chosen.) Repeated replacement of monophyletic bifurcations in a post-order traversal [10, page 319] of the gene tree implies that a whole subtree, where a gene follows species phylogeny, can be summarized into a single leaf. Post-order traversal ensures that no bifurcation is considered for replacement before its underlying bifurcations have been processed, see Fig. 3.

### Rule 2. Paraphyletic compression (Fig. 4)

**Figure 4:**
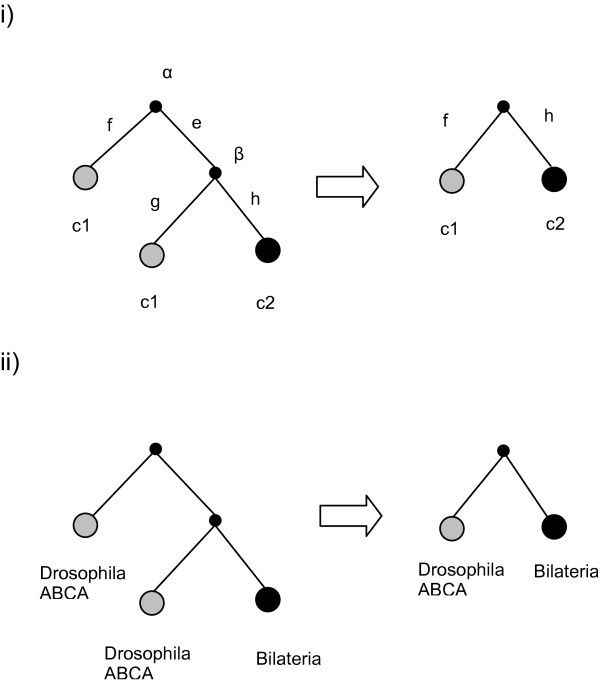
**Paraphyletic compression**. i) Edge *e *is considered paraphyletically redundant and is deleted, and *f *and *g *are summarized into a single edge. ii) Example of a paraphyletic compression using the ABC protein example.

The goal of paraphyletic compression is to allow a further limited amount of simplification in some parts of the gene tree even if species phylogeny is not followed. An outgoing edge *e *of a bifurcation *α*, leading to a bifurcation *β*, is *paraphyletically redundant *if the other outgoing edge *f *of *α *leads to a leaf with exactly the same label as *one *of the outgoing edges *g *and *h *of *β*. The other outgoing edge of *β *may lead to a node with a different label (see Fig. 4). In systematics, a *paraphylum *is defined as an incomplete monophylum, represented by the remainder of a subtree of the species tree caused by ignoring a subsubtree. In our case, *f *and *g *correspond to a paraphylum, and *h *corresponds to the ignored subsubtree. However, only redundant paraphyla are considered for simplification, limiting simplification to those cases where *f *and *g *lead to leaves with the same label, and replacing the paraphyletically redundant edge *e*, together with edges *f *and *g*, by *f*. (An example involving ABC proteins is mentioned below, ABC example part **(7)**.)

Both theoretical and empirical considerations support the deletion of a limited amount of paraphyly to simplify a gene tree. On one hand, such paraphyly is often caused by well-known branch attraction artifacts in phylogenetic reconstruction, in at least two ways. First, *g *and *h *may be long edges that got attracted [11]. Second, *g *and *h *may be short edges that got attracted, held together by ancestral character states (symplesiomorphies, [12]). In any case, the correct grouping features a bifurcation joining *f *and *g*. On the other hand, we made the empirical observation that large gene trees feature many cases of series of "orphan" genes splitting off one by one at the root of a subtree. However, according to our background knowledge, they should have been included together in one subsubtree at the root of that subtree.

### Rule 3. Treating species as equivalent

*TreeSimplifier *finally supports tree simplification by leaf re-labeling. For example, the user may unify all yeast species by giving them a single label "Yeast". Replacing the different yeast species designations in the gene tree by a single common one, and truncating the species tree so that the new designation becomes a leaf, all bifurcations leading to two leaves labeled "Yeast" can be compressed by monophyletic compression. In other words, all yeast species are now treated as equivalent, and subtrees consisting of these yield a single leaf if the genes are classified consistently. (If the unifying label were *not *a leaf label in the species tree, it would be considered a group label, so that the preconditions for monophyletic compression would not be met.)

### ABC protein example

The ABC gene/protein family [13,14] serves as an example for the method presented in this study.

(1) A typical HUGO gene name is *ABCA6*. Here, "ABC" is the stem symbol of the ABC gene family. In Table 2, gene *ABCA6 *belongs to the ABCA subfamily and it makes up the single-member ABCA6 subsubfamily. The mouse *Abca8b *together with *Abca8a *(not shown) is forming the proper subsubfamily Abca8. The *F02E11.1 *gene of *C. elegans *is an unclassified ABC protein without any further information.

(2) In case of *ABCA6*, deleting the stem symbol leaves 'A6'. 'A' is taken as the subfamily letter. The number ('6') that follows is taken as the subsubfamily number. An example for a gene with missing gene classification is the *F02E11.1 *gene of *C. elegans*, for which even the stem symbol is missing. An example for a gene with an incomplete gene classification is a *D. melanogaster *gene annotated as *ABCC*.

(3) An example of an ABC subtree that has a mix of genes with HUGO names and genes with missing or incomplete classification is shown in Fig. 1, panel iv. Here, the longest gene classification is *ABCC10*, and the other gene names are all prefixes of this classification. The classification of the *Fugu *and *Anopheles *genes is missing. Therefore, the subtree is consistently classified and the other genes can all be considered to be *ABCC10 *genes, and compression can be carried out under this assumption. In contrast, neither *ABCA *and *ABCC *nor *ABCC1 *and *ABCC2 *would feature a consistent classification. As another example, if *Abca8b *is the longest classification in a subtree, a consistent classification allows only *ABC, ABCA, ABCA8 *as well as *Abca8b *as well as any gene name not starting with 'ABC'.

(4) An example of a subtree without a consistent classification is given in Fig. 1, panel v. Here, the gene *Abcc2 *from subsubfamily C2 of mouse (in the subsubtree to the left) is probably misplaced because the subtree to the right features subsubfamilies C1, C2 and C3.

(5) To classify consistently all genes which share the same subsubfamily such as *ABCA8*, ignoring the next level, and, therefore, classifying *Abca8a *and *Abca8b *consistently, we instruct the software to truncate the classifications after the subsubfamily level (that is, we truncate after level 2).

(6) A simple example for an application of monophyletic compression to a subtree of the ABC gene tree is shown in Fig. 2, panel iii.

(7) A simple example for an application of paraphyletic compression to a subtree of the ABC gene tree is shown in Fig. 4, panel ii.

### Gene tree implementation and visualization

The visualization component of our tool employs the hyperbolic tree implementation *Hypertree *[15]. Thus, our tree is represented by a double-linked tree structure that interfaces to the *Hypertree *visualization package using Java. However, it can be easily modified to interface to any other phylogenetic tree visualization package written in Java, such as Walrus [16]. This may be important for non-academic users in the US, since commercial use of *Hypertree *may be problematic there, cf. [15]. The simplification rules are separated from the rest of the software using inheritance from a Java abstract class. Several generic methods are part of this class; among these is the application of a rule to the tree. To integrate new rules without recompiling the software package, a runtime loader for rules is provided. The application generates output trees in Newick format, reversing the operation of the parser and using the fields for the classification, the species name and the discriminator to generate the leaf labels.

### Graphical user interface

**Figure 5:**
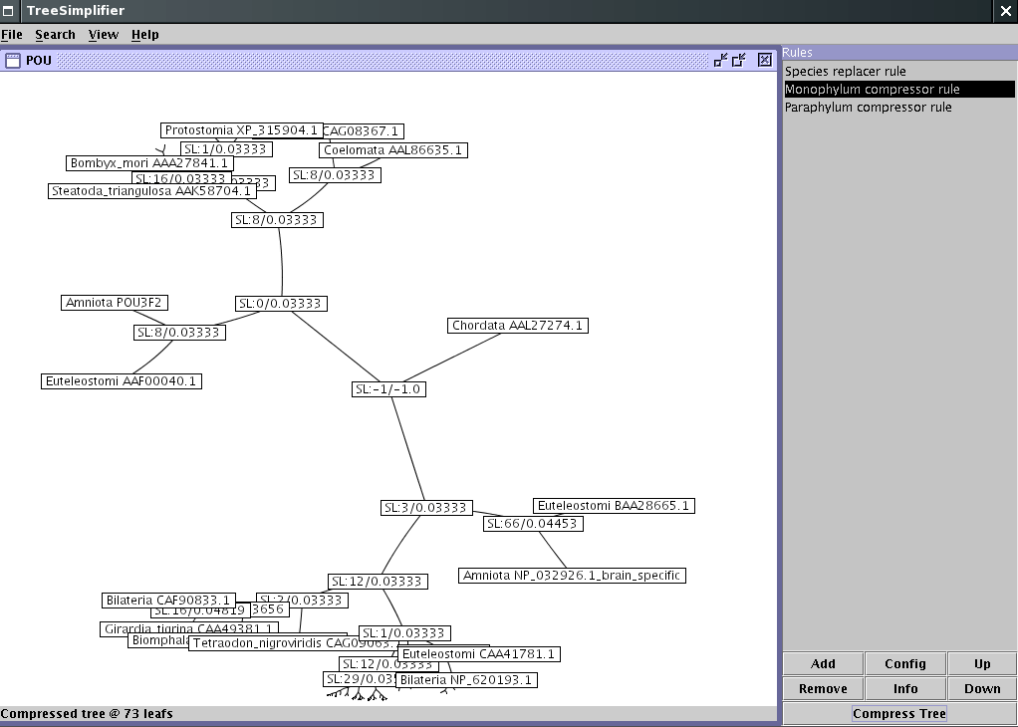
**Screenshot of the TreeSimplifier application**. Here, a POU transcription factor tree is compressed to 74 leaves, by treating all species from the *Amniota *clade as equivalent. On the left, the tree is visualized in a hyperbolic way. On the right, the rule manager panel is shown. It includes a "species replacer rule" that relabels all *Amniota *species, a "monophylum compressor rule" that uses the NCBI tree modified so that *Amniota *is a species leaf, and a "paraphylum compressor rule".

Using the awt and swing frameworks (part of the Java distribution), a graphical user interface was developed, including a „Simplification Rules" menu that dynamically lists all the rules available, giving the user the ability to apply them to the current tree in a specified order, and to add new ones. A text field lists the number of leaves in the current tree. It is possible to switch back and forth between the original and the simplified tree, and to search for certain labels in both trees. Nodes containing matching labels are centered. A screenshot is given by Fig. 5.

## Results and Discussion

We report use of the *TreeSimplifier *for the compression of a tree of ABC proteins [13,14] (1,138 sequences) and a tree of POU transcription factors [17] (185 sequences). The resulting simplified gene trees are 65% and 47% smaller, respectively. They are much easier to comprehend, even though their essential information content is preserved.

### Application to an ABC gene tree

The TreeSimplifier application was at first developed to perform simplifications of gene trees generated by automated pipelines such as the RiPE (Retrieval-induced Phylogeny Environment) analysis pipeline [1], [18]. RiPE performs an evolutionary analysis of a protein family, e.g. the ABC proteins of 20 model organisms. Generally speaking, ABC (ATP-Binding-Cassette) proteins are a family of proteins that transport substances across membranes, powered by ATP (adenosine triphosphate) hydrolysis. Most eukaryote ABC proteins can be classified into a number of subfamilies (ABCA to ABCG, [13,14]), which can be further divided into subsubfamilies. Most subsubfamilies consist of a single member. Functional ABC proteins (so-called full transporters) consist of two similar ATP-binding cassettes (symbols a, *α*), and two less similar transmembrane regions (symbols t, *τ*), in the order at*ατ*, or in reverse order ta*τα*. The two fragments are also called "halves" and they are due to an internal tandem repeat of the two domains. A detailed discussion of how the tandem repeat structure affects tree simplification is given in the appendix [see appendix 1].

### Comparison of a manually compressed tree with an automatically compressed tree

First, the output of *TreeSimplifier *was compared to a manual compression of a large gene tree of ABC proteins generated from 20 model organism proteomes by RiPE [1], and differences between both simplified trees were investigated. Towards this aim, we took the full tree, the cartoon tree and the species tree published by [1]. Simplification rules for the cartoon tree were published together with the tree, and they are summarized in the appendix [see appendix 2]. In fact, formalization of these rules yields the three rules described in the "Implementation" section, that are *monophyletic compression *(replacing a consistently classified gene subtree that follows species phylogeny by a single leaf), *paraphyletic compression *(deleting certain 'paraphyletically redundant' edges and subtrees) and *treating species as equivalent*. In the ABC example, the species treated as equivalent are the two yeast, plant (and fly) species, respectively, and the ABC classification hierarchy is ignored after the subsubfamily level. (In the automatic simplification, no difference was made between the flies *Anopheles *and *Drosophila*, in particular not for single-species leaves. They are distinguished, however, in the manual cartoon tree.) Overall, a tree of 1,138 leaves is compressed to a tree of 397 leaves, in 350 milliseconds on an Intel Pentium 4 (3 GHz).

**Figure 6:**
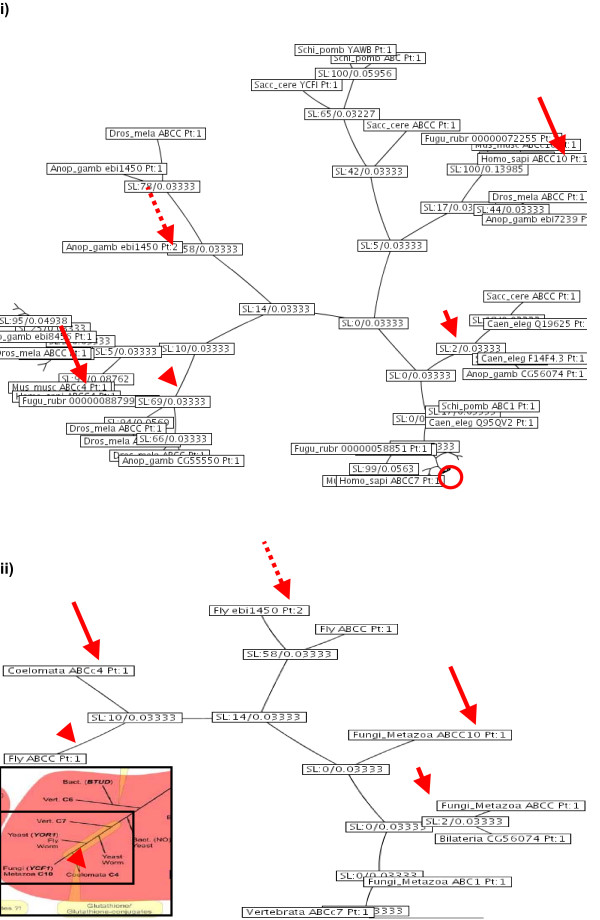
**Simplification of an ABC gene tree, main example**. **i) **Part of the original ABC gene tree, visualized by the *TreeSimplifier *GUI. On the very left, an arrow marks mouse ABCc4, which is part of the ABCC4 subtree, first internal repeat ("Mus_musc ABCc4 Pt: 1"; "Pt: 1" denotes the position of the repeat, distinguishing the first and the second "half" in case of ABC proteins [see appendix 1). On the very right, another arrow marks human ABCC10 from the ABCC10 subtree, again of the first internal repeat. In between, a subtree of fly sequences is found, and this subtree also features a gene fragment that is the second domain repeat in the *Anopheles *sequence ebi1450 (dashed arrow). In the bottom-right, subtrees including sequences from yeast, worm and fly are shown first (small arrow), followed by ABCC7 and the rest of the tree (circle). Subtree labels ("SL") denote the bootstrap value and branchlength associated with internal nodes. **ii) **Part of the simplified tree automatically provided by *TreeSimplifier*, and a manual simplification (inset, for comparison, taken from 1). In the automatic simplification, no difference was made between *Anopheles gambiae *and *Drosophila melanogaster*. Thus, the ABCC4 subtree follows species phylogeny up to the Coelomata (Vertebrata and fly, see the top-left arrow), and the subtree bottom left in the original tree (triangle arrow) contributes another edge labeled "Fly ABCC Pt: 1". Since different internal repeats (first half versus second half) are not treated as classified consistently in case of ABC proteins, "Pt: 1" and "Pt: 2" cannot be unified (see dashed arrow), although they are glossed over in the manual simplification; "Pt: 2" is most likely a mis-annotation by the RiPE pipeline. The ABCC10 subtree follows species phylogeny, and it is replaced by the single leaf "Fungi_Metazoa ABCC10 Pt. 1" (see the arrow at the very right). There are some more subtrees on the bottom right, namely (Fungi/Metazoa, Bilateria) (small arrow), Fungi/Metazoa and Vertebrata ABCC7. The manual compression (inset) glosses over some fly sequences (triangle arrow) in case of ABCC4, because it treats any mix of *Drosophila *and *Anopheles *as a single fly contribution to the gene tree, which is then included, together with Vertebrata ABCC4, in the leaf "Coelomata ABCC4". Moreover, human simplification uses more specific labels such as "Yeast/Worm" instead of "Fungi/Metazoa". TreeSimplifier took much less than a second to derive the entire cartoon tree of 397 leaves.

**Figure 7:**
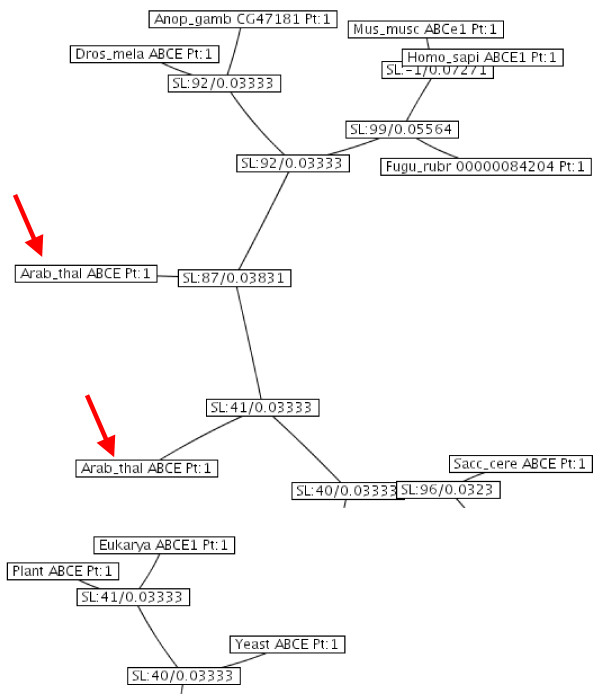
**Simplification of an ABC gene tree, example for non-commutativity of rules**. The original tree is shown on top, the automatically simplified tree at the bottom. In this example, applying paraphyletic compression first (simplifying the two marked nodes into one) and monophyletic compression later would have resulted in a further simplified subtree only composed of "Eukarya ABCE1 Pt: 1" and "Yeast ABCE Pt: 1". In other words, monophyletic and paraphyletic compression are not commutative.

**Figure 8:**
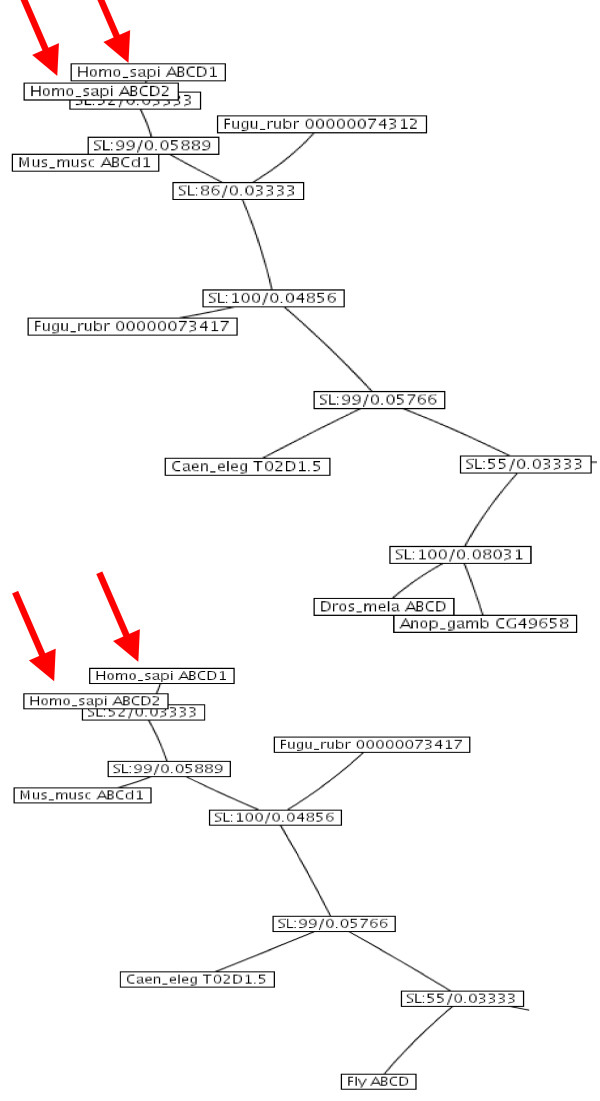
**Simplification of an ABC gene tree, example for erroneous input data that cannot be corrected**. The original tree is shown on top, the automatically simplified tree at the bottom. Here, the manual simplification in [1] went much farther by treating human ABCD1 and ABCD2 (arrows) as classified consistently since both are due to a recent duplication. In fact, the existence of a single mouse homolog indicates that in fact, ABCD1 and ABCD2 should be denoted ABCD1a and ABCD1b. In that case the automatic simplification would match the manual one since the classification hierarchy is ignored after the subsubfamily level, compressing the entire subtree to a single "Bilateria ABCD" leaf, except for the fly sequences at the root of the subtree.

**Figure 9:**
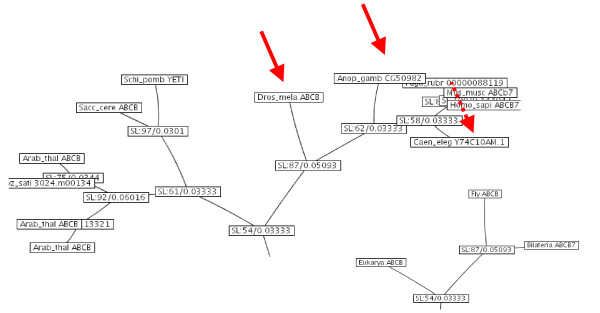
**Simplification of an ABC gene tree, example for detection of human oversight due to automation**. The original tree (left) gives rise to a simplified tree (right) in a straightforward way by monophyletic compression, treating yeast sequences as well as plant sequences alike, and applying paraphyletic compression to the leaves marked by arrows. The human simplification in [1] just lists a single bifurcation of "Plant (ATM3)/Yeast (ATM1)" and "Bilateria B7", not considering the position of the worm sequence (dashed arrow) due to human oversight.

**Figure 10:**
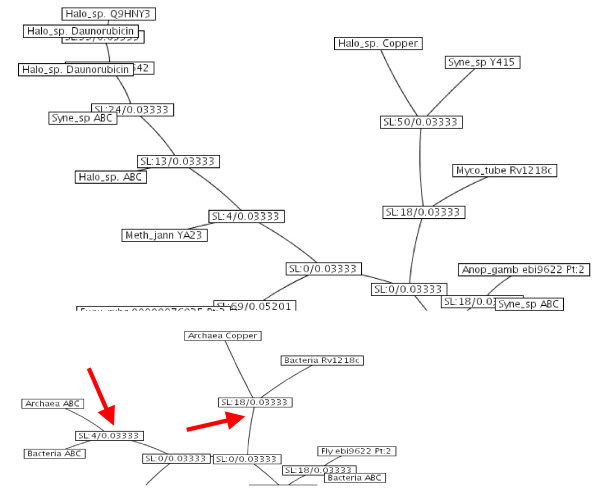
**Simplification of an ABC gene tree, example of 'creative' further simplification that is not automated**. The original tree is shown on top, the automatically simplified tree at the bottom. Manual simplification in [1] goes one step further in applying a generalized version of the paraphyletic compression rule: Since both subtrees (arrows) consist of a bifurcation of archaea and bacteria, they are merged into one.

Compression is detailed for five subtrees in Figs. 6 to 10. *TreeSimplifier *provided almost the same simplification result as the manual analysis. The manually compressed tree was occasionally further simplified, in a way that made sense from the biological point of view, but which violated the formal simplification rules (see Fig. 6). In one case, the order in which the rules are applied turned out to be important (Fig. 7). Moreover, manual compression corrects erroneous input data very easily while an algorithm cannot do so (Fig. 8). In turn, human processing introduces some errors (Fig. 9). In some cases, creative application of rules was made in the manual compression (Fig. 10). We were able to explain all remaining differences between the automatic and the manual simplification in one of the five ways just described (data not shown). Thus, automation by *TreeSimplifier *saves many hours of manual work and it yields a result of comparative quality.

### POU transcription factor tree

A further example of a gene tree simplification, using POU transcription factors [17], is given in the supplementary material [see Additional file 1].

### Information loss by simplification

The qualitative nature of the information loss is different for each of the three rules we introduced. Using the first rule (monophyletic compression), a group leaf is created and labeled with the designation of the group of species to which the proteins did belong, and the information is lost on "which species exactly" were represented. Using the second rule (paraphyletic compression), we instead ignore some of the information about the structure (topology) of the gene tree. The effect of the third rule (species equivalence classes) combines both losses of information; tree topology as well as species distribution are glossed over as long as the species are in the set defined as equivalent by the user.

### Future work

Future work includes consideration of bootstrap confidence values and branchlengths, a command-line interface, export of vector graphics, coloring of labels (e.g. by subfamily) and a generic user-friendly rule editor.

## Conclusion

An automatic simplification tool for gene trees was described that is fast, effective, and less prone to errors than manual simplification. Given the huge amount of data generated by genome sequencing efforts, our tool should be valuable as an automatic aid for phylogenetic studies of protein families.

## Availability and requirements

• Project name: TreeSimplifier

• Project home page:



• Operating system(s): Platform independent

• Programming language: Java

• Other requirements: Java 1.4.2 or higher

• License: GNU GPL/MIT license (Hypertree library)

• Any restrictions to use by non-academics: None; a patent on hyperbolic tree visualization (this type of visualization is used by the GUI to display the trees) is believed to be invalid, but may nevertheless pose a problem to commercial users in the US (see also [15]).

## Abbreviations

• ABC, ATP-binding Cassette

• POU, acronym derived from three mammalian transcription factors, the pituitary-specific Pit-1, the octamer-binding proteins Oct-1 and Oct-2, and the neural Unc-86 from *Caenorhabditis elegans*

• HUGO, Human Genome Organisation

• EST, Expressed Sequence Tag

• RiPE, Retrieval-induced Phylogeny Environment

• NCBI, National Center for Biotechnology Information

## Authors' contributions

PLL, MM and CS wrote the software. MM wrote the website and the documentation. SL contributed parts of the manuscript and was involved in the design of the study. GF conceived the study and wrote the manuscript. All authors read and approved the final manuscript.

## Appendix 1. ABC proteins [13,14], HUGO gene names and internal repeat arrangements

The two fragments, or "halves" (at/ta and *ατ*/*τα*; see *Results and Discussion*) of many human ABC transporters are very similar. For a given ABC subfamily, they are most similar amongst themselves (e.g., the ABCA first halves cluster together, just like the ABCA second halves do). They are *not *most similar for each member of a subsubfamily, nor do they globally cluster together in two big subtrees, one per half [1]. Therefore, it is assumed that a tandem gene duplication took place at the origin of most full-transporter subfamilies, yielding ABC full transporters that consist of two halves. ABC gene trees include two fragments per full transporter, and simplification must consider which half we are dealing with. For example, a subtree of ABCA first halves should not be classified consistently (see the "Implementation" section), if a single sequence from the second half is included. In terms of HUGO gene names, a *hypothetical *naming scheme considering gene fragments due to (tandem) duplications would include the corresponding classification information at the appropriate level of the family hierarchy, such that ABCA1 would be the subsubfamily of all first halves, and ABCA**1**A would be the **first **half of what is known as ABCA1, and ABCA**2**C would be the **second **half of what is known as ABCA3, etc, see Table 3.

More generally, we want to deal with cases where a gene sequence consists of subsequences that are repeated such that the gene must be represented by several leaves in the gene tree that each correspond to one of the internal repeats (gene fragments). These need to be analyzed phylogenetically as separate units. However, as we have seen, HUGO gene names do not include any information with respect to the duplication history of the main repeat arrangement. While the user may encode her expert knowledge in a set of appropriate names for the gene fragments as in the hypothetical gene names of Table 3, our tool offers a workaround for the two most likely cases: Either the issue is ignored and gene fragments are classified consistently no matter what their position (first half, second half, etc) is, thus assuming that they should be classified consistently since all duplications arose at the lowest level of the classification. Or, gene fragments from different halves are never classified consistently, thus assuming that all duplications arose at a higher level. As described in the "Implementation" section, this functionality is implemented by parsing in a "discriminator" string that may be present in leaf labels, and, by default, using this string to establish that whenever it is different, the leaves cannot be classified consistently.

## Appendix 2. Simplification Rules for manual gene tree compression

The ABC gene tree obtained by RiPE was simplified manually by the following set of rules (cf. [1], yielding Figure 2 in [1]). The automation of these rules is accomplished by *TreeSimplifier*.

1. *Monophylum compression*. Subtrees with sequences classified consistently that follow the species phylogeny, or that belong to a single species, were replaced by a single label designating the (group of) species to which they belong.

2. Unclassified sequences mixed with classified ones are assumed to have the same label as the classified ones, and they are ignored except that they contribute their species designation to the labels.

3. We distinguish *Anopheles gambiae *and *Drosophila melanogaster *as single leaves. But if sequences from both species occur in the same subtree, the annotation is "Fly".

4. We do not distinguish *Arabidopsis thaliana *and *Oryza sativa *proteins. Both species are designated as "Plant".

5. We do not distinguish *Saccharomyces cerevisiae *and *Schizosaccharomyces pombe*. Both species are designated as "Yeast".

*Paraphylum compression*. As long as consecutive edges in a subtree make up a backbone to which subsubtrees are attached that feature sequences classified consistently and that belong to the same species/clade, these edges are deleted and a single subtree that subsumes all subsubtrees is introduced.

## Supplementary Material

Additional File 1 Supplementary Material. POU transcription factor gene tree simplification. Click here for file

## Figures and Tables

**Table 1:** Main Features of the TreeSimplifier Software.

Input	A gene tree and a species tree. At least some of the gene names follow HUGO nomenclature (e.g. ABCC4, S100A12, POU5F1). All gene names must be supplemented with the name of the species in which the gene is found.
Output	A simplified gene tree. In particular, a subtree of the gene tree such as ((human ABCC4, mouse Abcc4), fugu ABCC) is simplified to a single node "vertebrate ABCC4", because ((human, mouse), fugu) corresponds to a monophylum in species phylogeny, and gene names are consistent.

Further simplification output	Further simplified gene tree: paraphyletic tree substructures such as (fly gene, (fly gene, vertebrate genes)) may be simplified to (fly gene, vertebrate genes), if gene names are consistent. Species may be treated alike: subtrees such as (((drosophila gene, anopheles gene), drosophila gene), (anopheles gene, anopheles gene)) may be simplified to a single node "fly genes" if both flies are to be treated alike and gene names are consistent.

GUI extras	Hyperbolic tree browsing, searching for text in the tree.

**Table 2:** Examples of deflines, and corresponding "canonical" leaf labels. Three genes are used as examples. Their deflines and the corresponding canonical labels are given. The canonical labels were generated from the deflines by RiPE [1], [18]. To obtain a useful tree simplification, at least some of the gene names should follow HUGO-based gene classification. For these, the subfamily letter following the HUGO stem symbol is given in bold, the subsubfamily number is underlined, and the subsubsubfamily letter (if any) is given in italics. The species name in bold comes next, followed by a discriminator that is underlined. HUGO classification is missing in the third example. In this case, we will tacitly assume a classification that is consistent, given the gene tree.

Label 1	|ABC**A**6|_ [**Homo_sapiens**]_ {part1}
Defline 1	NP_525023|__EMBL:Q8N139;Q8N856|ENSEMBL:ENSP00000284425 Tax_Id = 9606 ABC transporter ABCA6 [Homo sapiens]

Label 2	|Abc**a**8*b*|_ [**Mus_musculus**]_ {part1}

Defline 2	REFSEQ_NP:NP_038879|__EMBL:Q8K440|ENSEMBL:ENSMUSP00000018946; ENSMUSP00000020948 Tax_Id = 10090 Ensembl_locations(Chr-bp): 11-110773415;11-110788088 ATP-binding cassette transporter sub-family A member 8b [Mus musculus]

Label 3	|F02E11.1|_ [**Caenorhabditis_elegans**]

Defline 3	O16574 (O16574) F02E11.1 protein [Caenorhabditis elegans]

**Table 3:** Hugo gene names and gene phylogeny in case of ABC proteins with tandem repeats. If the two ABCA halves had split in evolution and been observed at different genomic loci, the hypothetical gene name would probably have been adapted as HUGO gene name.

	Stem	Subfamily level	Subsubfamily level	Subsubsubfamily level
Example 1: ABCA1, first half, should be ABCA1A				

Gene classification following gene phylogeny	ABC	A	(**first **half)	1

HUGO gene name	ABC	A	(not recognized)	1

Hypothetical gene name following gene phylogeny	ABC	A	**1**	A

Example 2: ABCA3, second half, should be ABCA2C				

Gene classification following gene phylogeny	ABC	A	(**second **half)	3

HUGO gene name	ABC	A	(not recognized)	3

Hypothetical gene name following gene phylogeny	ABC	A	**2**	C
