# Supplementary material for: Simplifying gene trees for easier comprehension
Source: BMC Bioinformatics. 2006 Apr 27;7:231. doi: 10.1186/1471-2105-7-231 (PMC1473203; doi:10.1186/1471-2105-7-231)
Supplement: Additional File 1 — Supplementary Material. POU transcription factor gene tree simplification. [file 1471-2105-7-231-S1.doc]

**Supplementary Data:**

# **Simplifying gene trees for easier comprehension**

Paul-Ludwig Lott^1,2,§^ Marvin Mundry^1,2,3,§^ Christoph Sassenberg^1,2,§^ Stefan Lorkowski^4,5^, Georg Fuellen^1,3,6,*^

1 Division of Bioinformatics, Biology Department, University Münster, Schlossplatz 4, 48149 Münster, Germany

2 Institut für Informatik, Fachbereich Mathematik und Informatik, Einsteinstr. 62, 48149 Münster, Germany

3 Department of Medicine, AG Bioinformatics, University Münster, Domagkstrasse 3, 48149 Münster, Germany

4 Leibniz-Institute of Arteriosclerosis Research, University Münster, Domagkstrasse 3, 48149 Münster, Germany

5 Institute of Biochemistry, University Münster, Wilhelm-Klemm-Str. 2, 48149 Münster, Germany

6 Institute of Mathematics and Computer Science, University Greifswald, Jahnstrasse 15a, 17489 Greifswald, Germany

^§^ These authors contributed equally to this work.

**POU transcription factor tree.** Using the *TreeSimplifier* tool described in the main paper we simplified a gene tree (Fig. S1_) of POU transcription factors (see e.g. [17]), resulting in the gene tree shown in Fig. S2_. The simplified tree has 96 leaves, while the original tree has 185 leaves. The latter was generated using the RiPE pipeline [1], searching the entire NCBI NR (non-redundant) database with a profile of POU5F1 sequences from several organisms. Moreover, HUGO gene names were added to the deflines of the human POU proteins. (POU5F1 is also known as the Oct3/Oct4 transcription factor.) To guide monophyletic compression, we used the entire NCBI taxonomy as the species tree, converted to Newick format, and taking care of nodes with a single leaf. (For example, the node “*Homo sapiens*” with the single leaf “*Homo sapiens* *neanderthalensis*” is converted to the bifurcation (“*Homo sapiens*”, “*Homo sapiens neanderthalensis*”). The putative phylogeny of POU factors is much easier to recognize in the simplified tree than in the original tree, and species names that are not well known such as *Mesocricetus auratus* in case of the POU3F4 subtree are often subsumed by names for well known groups of species such as “Coelomata”.

**Figure S1_.** **Original POU gene tree.** A simplification of this tree of POU transcription factors can be found in Fig. S2_. NJPLOT (Perrière G, Gouy M: WWW-query: an on-line retrieval system for biological sequence banks. Biochimie 1996, 78:364-369) was used to generate the Figure.

**Figure S2_. Simplified POU gene tree.** The original tree of POU transcription factors can be found in Fig. S1_. HUGO gene names such as POU5F1 start with “POU”, followed by subfamily designation. The letter “F” that is found thereafter is invariant, and it is followed by the single-member subsubfamily number. If nodes are compressed but HUGO gene names are missing in an entire subtree, the first gene name is chosen. Branches are labeled by resampling (bootstrap) support given as percentages based on 1000 replicates. NJPLOT was used to generate the Figure.

**
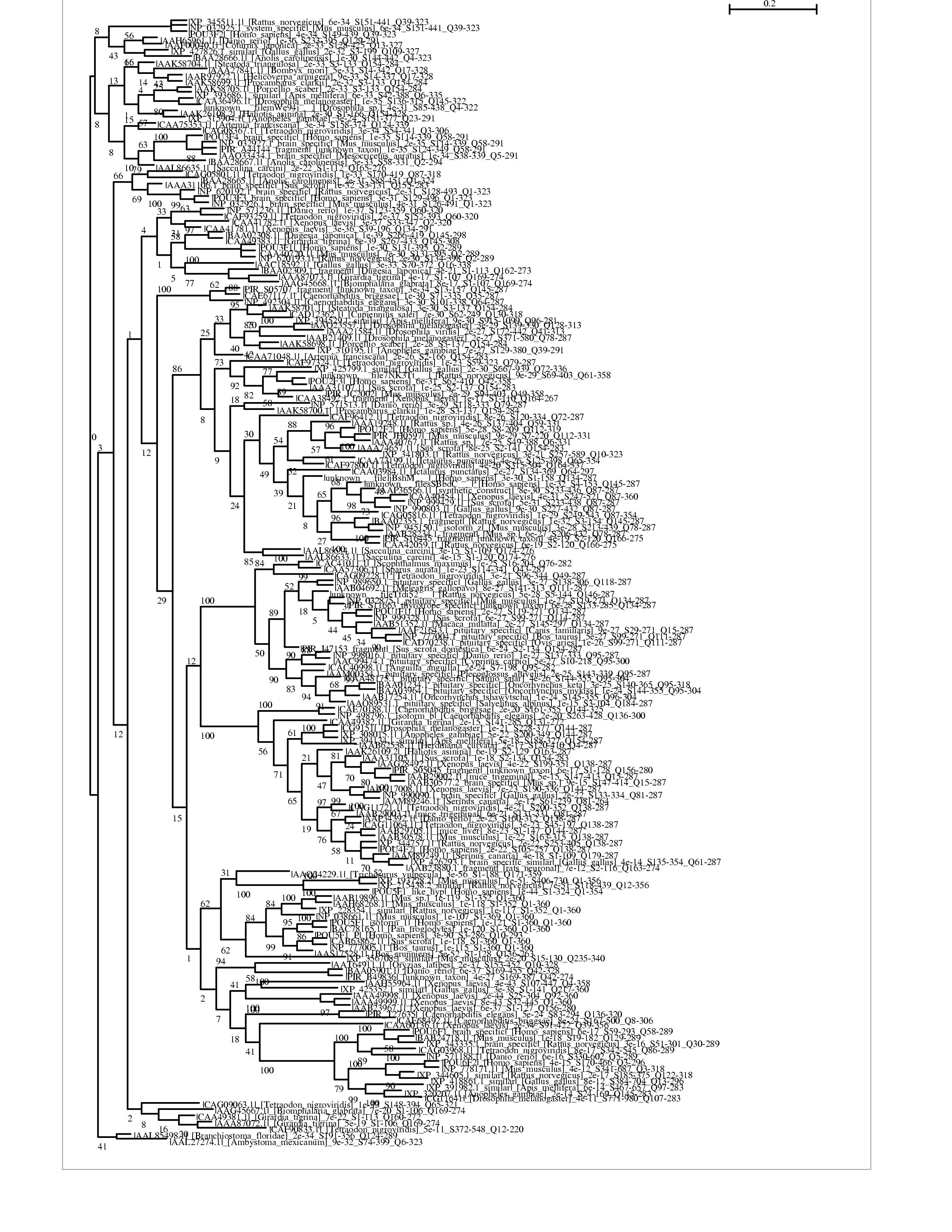
Figure S1_.** **Original POU gene tree.**

**
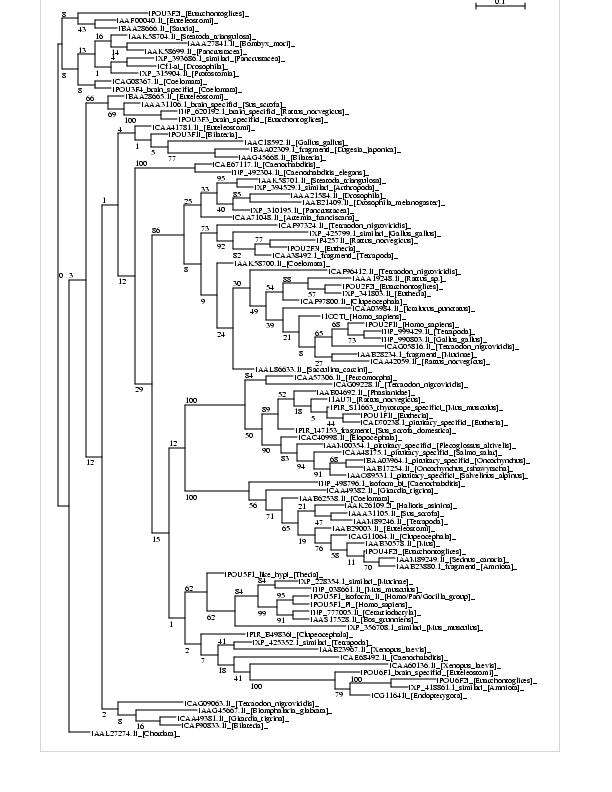
**

**Figure S2_.** **Simplified POU gene tree.**

POU3

POU2

POU1

POU4

POU5

POU6
